# Pericardial Effusion as an Unusual Complication of Laparoscopic Ventral Hernia Repair: A Case Report

**DOI:** 10.7759/cureus.105093

**Published:** 2026-03-12

**Authors:** Matthew J Scearbo, Yazan Abuawad, Sydney Elness, Alejandro Biglione

**Affiliations:** 1 Department of Medicine, Nova Southeastern University Dr. Kiran C. Patel College of Osteopathic Medicine (KPCOM), Tampa, USA; 2 Department of Internal Medicine, Wellington Regional Medical Center, Wellington, USA; 3 Department of Medicine, Nova Southeastern University Dr. Kiran C. Patel College of Osteopathic Medicine (KPCOM), Davie, USA

**Keywords:** epigastric hernia, inflammation, laparoscopic ventral hernia repair, pericardial effusion, pericarditis

## Abstract

Pericarditis is a well-recognized complication of cardiac surgery and myocardial injury; however, it is rarely reported following non-cardiac procedures. The mechanisms underlying postoperative pericardial inflammation in such cases remain poorly defined. This case report is about a patient who developed a pericardial effusion two weeks after repair of a ventral hernia in the midline with a laparoscopic technique. No evidence of infection, malignancy, autoimmune disease, or myocardial injury was found. This case report discusses the possible etiologies, pathophysiologic mechanisms, and treatment of pericardial effusion after non-cardiac surgery.

## Introduction

Acute pericarditis is inflammation of the pericardial membrane. It is diagnosed when two out of four criteria are met: pleuritic chest pain, diffuse ST elevation and PR depression on ECG, pericardial effusion, or pericardial friction rub [1]. Most cases of pericarditis in developed nations (80-90%) are deemed idiopathic and presumed to be of viral etiology. Common identifiable causes of pericarditis are neoplastic syndromes, autoimmune conditions, metabolic derangements, and post-cardiac injury syndromes due to myocardial injury or cardiac surgeries. Pericarditis occurs in 20-30% of cardiac surgeries [1]. Direct trauma to the pericardium is expected to produce an inflammatory response. However, clinically significant pericardial inflammation and effusion after non-cardiac surgery are rarely reported in the current literature. We present a case of acute pericarditis two weeks after laparoscopic repair of an incarcerated epigastric hernia. Known causes of pericardial inflammation, as previously mentioned, were ruled out. In the absence of a viral prodrome, it was reasonable to presume the patient's recent abdominal surgery as a contributing factor. Systemic inflammation and immune activation due to invasive surgery and prosthetic materials are mechanisms to be considered.

## Case presentation

A 65-year-old woman complained of sudden onset, central pressure-like chest pain radiating to her shoulders, neck, and teeth that woke her up from sleep in the middle of the night. The pain was pleuritic, worsened with deep inspiration and leaning forward, and associated with mild dyspnea. The patient self-administered acetaminophen without relief. The patient denied palpitations, cough, nausea, vomiting, or dizziness. No recent illness or travel was reported by the patient.

Her past medical history included anemia, anxiety, asthma, diabetes, hypertension, and breast cancer treated with lumpectomy and radiation in 1998. Two weeks earlier, she underwent laparoscopic repair of an incarcerated upper midline ventral hernia. The hernia was incarcerated and repaired via a laparoscopic technique. Other surgical history included cholecystectomy, abdominoplasty, gastric bypass, and two C-sections, all remote operations with no recent surgical interventions.

She presented to the emergency room (ER) a few hours after symptom onset, and her vitals upon arrival were as follows: temperature (T), 98.8°F; heart rate (HR), 76 beats per minute; respiratory rate (RR), 14 breaths per minute; blood pressure (BP), 148/75 mmHg; and peripheral oxygen saturation (SpO_2_) 97% on room air. Vitals remained stable without acute changes during her admission.

On physical examination, the patient was awake, lying comfortably in bed, with no acute cardiopulmonary distress, non-toxic, and non-diaphoretic. She answered questions appropriately, alert and oriented to person, place, and time. The neck was supple, with a full range of motion and no jugular vein distention (JVD). Lung examination showed no wheezing, rales, or rhonchi. No accessory muscle usage or conversational dyspnea was reported. Heart sounds were normal S1-S2, with regular rate and rhythm (RRR), and no murmurs or friction rub were appreciated. The abdomen was soft, non-tender, non-distended, without guarding or rebound tenderness. No focal neurological deficits were reported. In addition, no rashes, skin lesions, petechiae, purpura, or edema were observed. No cyanosis, clubbing, or gross deformities were present.

**Figure 1:**
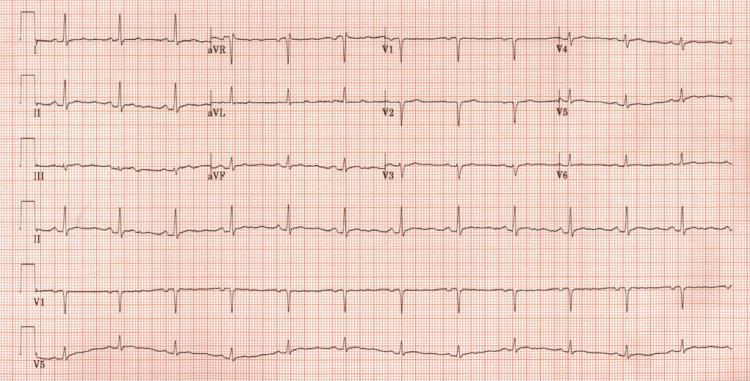
Electrocardiogram The electrocardiogram shows a normal sinus rhythm, a normal axis, and no acute ST changes.

Labs were drawn, and an EKG was obtained in the emergency department, which is shown in Table 1 and Figure 1, respectively.

**Table 1:** Laboratory Results Labs were notable for an elevated WBC count with thrombocytosis and an elevated CRP. "L" refers to low for the laboratory reference range. "H" refers to high for the laboratory reference range. WBC: white blood cells; RBC: red blood cells; Hgb: hemoglobin; Hct: hematocrit; MCV: mean corpuscular volume; MCH: mean corpuscular hemoglobin; MCHC: mean corpuscular hemoglobin concentration; RDW-SD: red cell distribution with-standard deviation; RDW-CV: red cell distribution with-coefficient of variation; MPV: mean platelet volume; Neut: neutrophil; Lymph: lymphocyte; Mono: monocyte; Eos: eosinophil; Baso: basophil; Immature gran: immature granulocyte; Sed rate: sedimentation rate; PT: prothrombin time; INR: international normalized ratio; aPTT: activated partial thromboplastin time; POC: point-of-care; AGAP: anion gap; BUN: blood urea nitrogen; Creat: creatinine; Est. creat.: estimated creatinine; A/G: albumin/globulin; T Bili: total bilirubin; Alk phos: alkaline phosphatase; AST: aspartate aminotransferase; ALT: alanine aminotransferase; eGFR Cr: estimated glomerular filtration rate calculated via serum creatinine (Cr); Hgb A1C: hemoglobin A1c; CRP: C-reactive protein; Pro BNP: pro-B-type natriuretic peptide; Lvl: level; Troponin TNIH: high-sensitivity Troponin I; Chol: cholesterol; Trig: triglyceride; HDL: high-density lipoprotein; LDL: low-density lipoprotein; VLDL: very-low-density lipoprotein; IFA LC: cytosolic liver antigen type-1.

Laboratory Measurement	Normal Values/Units for Institution	Day 1	Day 2
WBC	4.5-10.5 x 10^3/mcL	12.09 H	7.33
RBC	4.2-5.5 x 10^6/mcL	4.22	3.74 L
Hgb	12-16 gm/dL	12	10.7 L
Hct	37-47%	36.9 L	33.6 L
MCV	81-96 Femtoliters	87.4	89.8
MCH	27-34 pg	28.4	28.6
MCHC	32-36 gm/dL	32.5	31.8 L
RDW-SD	36-50 Femtoliters	47.9	50.1 H
RDW-CV	11-14.5%	14.90 H	15.10 H
Platelet	150-450 x 10^3/mcL	467 H	421
MPV	6.9-10.5 Femtoliters	9.3	10
Neut % Auto	40-77%	77.7 H	71
Lymph % Auto	24-44%	13.9 L	16.4 L
Mono % Auto	0-15%	6.9	8.3
Eos % Auto	0-10%	0.9	3.4
Baso % Auto	0-2%	0.9	0.5
Immature gran %	0-5%	0.3	0.4
Neut # Auto	1.8-7.7 x 10^3/mcL	9.38 H	5.2
Lymph # Auto	0.8-2.8 x 10^3/mcL	1.68	1.2
Mono # Auto	0.2-1 x 10^3/mcL	0.84	0.61
Eos # Auto	0.0-0.6 x 10^3/mcL	0.11	0.25
Baso # Auto	0.0-0.3 x 10^3/mcL	0.04	0.04
Immature grans # Auto	0.0-0.5 x 10^3/mcL	0.04	0.03
Sed rate	0-20 mm/hr	20	-
PT	9.7-11.7 seconds	10.8	-
INR	0.9-1.2	0.99	-
aPTT	24-35 seconds	27.1	-
D-Dimer	0.19-0.52 mg/L FEU	0.92 f H	-
Glucose level	74-106 mg/dL	120 H	117 H
POC glucose	75-110 mg/dL	-	-
Sodium	135-148 mmol/L	138	138
Potassium	3.6-5.2 mmol/L	4.4	4.4
Chloride	95-110 mmol/L	103	103
CO_2_	21-32 mEq/L	30	31
AGAP	5-15 mmol/L	9.4	8.4
BUN	7-18 mg/dL	21 H	20 H
Creatinine	0.61 mg/dL	0.53 L	0.61
BUN/creat ratio	-	40	33
Calcium	8.5-10.1 mg/dL	9.4	8.8
Albumin level	3.4-5.0 gm/dL	3.3 L	2.8 L
Total protein	6.4-8.2 gm/dL	7.3	6.1 L
A/G ratio	0.95-1.59	0.82 L	0.85 L
T Bili	0.0-1.0 mg/dL	0.5	0.7
Alk phos	45-117 IU/L	72	115 f
AST	15-37 IU/L	16	215 f H
ALT	12-78 IU/L	19	254 f H
Est. creat. clear. Cockcroft method	-	91.38 f	79.40 f
eGFR Cr	ml/min/1.73m^2	102 f	99 f
eGFR pediatric	-	Not Reported	Not Reported
Lipase Lvl	16-77 IU/L	26	-
Hgb A1C	3.8-5.6	-	5.9 H
CRP	0.0-0.3 mg/dL	4.01 H	-
Pro BNP	0-215	170	-
Troponin TNIH	0-53.7 ng/L	3.7 f/3.2 f	-
Chol	112-200 mg/dL	110 L	-
Trig	35-160 mg/dL	29.0 L	-
HDL	30-85 mg/dL	52	-
LDL calculated	100-129 mg/dL	52 L	-
HDL/chol risk	-	0.47 f	-
VLDL chol calc	mg/dL	6	-
Antinuclear antibodies, IFA LC	-	Negative f	-

**Figure 2:**
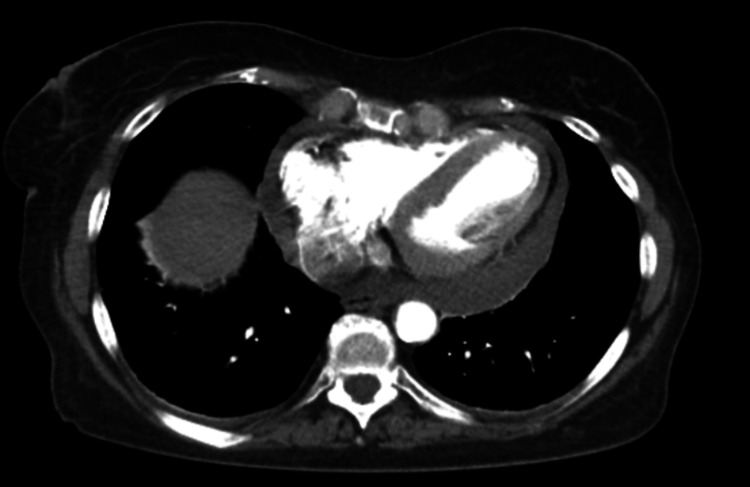
Axial CT Angiography of the Chest With Intravenous Contrast The CT shows moderate pericardial effusion. Pulmonary arteries are free of emboli, with no pleural effusions. No pathologically enlarged lymph nodes are observed. No thoracic aortic aneurysm or dissection is present. The lung parenchyma shows no consolidation or ground-glass opacities. CT: computed tomography.

**Figure 3:**
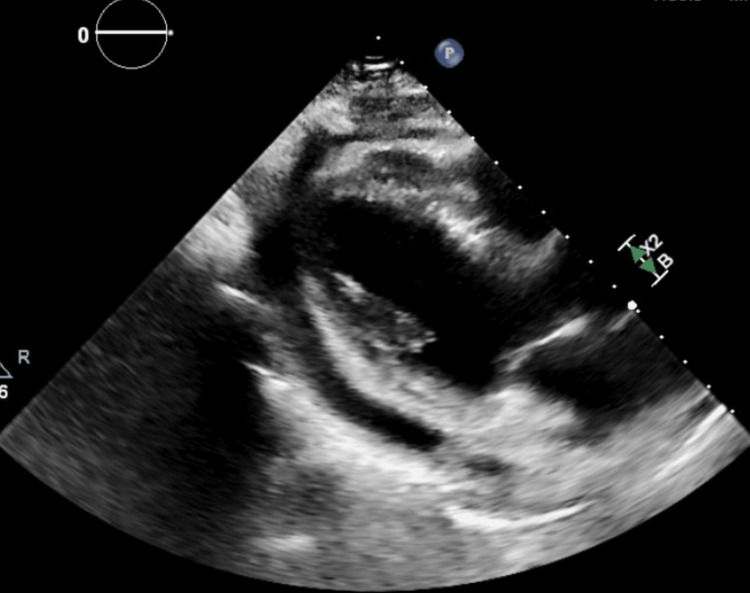
Echocardiogram Parasternal Long-Axis View The ECHO shows a moderate pericardial effusion measuring 12 mm without findings of tamponade physiology. Normal global systolic left ventricular function with ejection fraction estimated at 60-65% was noted. The right ventricular systolic pressure was 26 mmHg. ECHO: echocardiogram.

Upon admission, CT angiography of the chest and a transthoracic echocardiogram were performed. Images of these tests are shown in Figures 2-3.

Based on the presence of pleuritic chest pain and significant pericardial effusion, the diagnosis of pericarditis was made. Significant elevation of CRP supported the suspicion of an inflammatory process. No tamponade physiology was present on echocardiogram, and no signs of hemodynamic compromise were present on clinical assessment. Differential diagnoses included pericarditis of post-inflammatory, viral, or idiopathic etiology, malignant pericardial effusion, and radiation-induced pericardial disease. The patient was started on colchicine 0.6 mg BID daily with 600 mg ibuprofen TID. On admission day 2, her aspartate aminotransferase/alanine aminotransferase (AST/ALT) increased from 16/19 U/L on admission to 215/254 U/L (normal range 15-37/12-78 U/L). There was no clear explanation for the increase in liver enzyme activity, as one dose of colchicine would not be expected to cause a sudden transaminitis. However, colchicine was discontinued on day 2 due to the potential of colchicine to cause liver injury. The patient requested discharge due to total symptomatic improvement on admission day 2; therefore, labs and imaging were not repeated to treat her transaminitis or confirm resolution of pericardial effusion.

The patient's discharge plan included ibuprofen 600 mg TID for 10 days with instructions to repeat a comprehensive metabolic panel (CMP) outpatient and follow up with her primary care physician (PCP) and cardiology within one week. There has been no known recurrence of the patient’s pericarditis.

## Discussion

The recent 2025 American College of Cardiology (ACC) consensus clinical guidance on pericarditis lists no quantifiable incidence of pericarditis following non-cardiac procedures [2]. A few such instances of pericarditis or other pericardial complications have been reported after non-cardiac surgery. Laparoscopic repair of a ventral hernia has never been cited as a cause of pericarditis according to the current literature. The majority of cases, similar to the one presented, involve pericarditis or tamponade as a complication of diaphragmatic or hiatal hernia repair [3-7]. It is well documented that the mesh and tacks used for fixation in these procedures can cause significant inflammation, which is the proposed mechanism for these complications [5-8]. It is because there is just a thin part of the diaphragm separating the area of fixation from the heart in hiatal hernia repair [9]. Ventral upper midline abdominal hernia repair does not share this same relationship; therefore, the mechanism causing pericarditis in this case is less obvious. However, these cases help support the idea that a systemic inflammatory response due to the surgical materials used for fixation could have contributed to the pericarditis observed in the presented case. In addition, invasive surgery in general is known to cause dysregulated systemic inflammatory responses [10,11]. The patient’s history of radiation treatment for breast cancer may have predisposed the pericardium to developing this sequela in the context of systemic inflammation due to invasive surgery. This case contributes to the ongoing literature of complications associated with surgical materials used for hernia repair, and supports the idea that systemic inflammatory responses can arise following invasive procedures, leading to unexpected presentations.

## Conclusions

Pericarditis following non-cardiac surgery is rare. However, invasive procedures are capable of inducing systemic inflammation that may manifest as pericarditis in certain predisposed patients. Certain procedures, such as hernia repairs, use prosthetic materials known to cause inflammatory responses that may also contribute to this clinical picture. This case report emphasizes the need for increased awareness to promote early detection and treatment of this potentially problematic complication of ventral hernia repair.
